# Can total knee arthroplasty be safely performed in patients with chronic renal disease? An evaluation of perioperative morbidity in 2,686 procedures from a Total Joint Replacement Registry

**DOI:** 10.3109/17453674.2013.878829

**Published:** 2014-02-25

**Authors:** Alexander Miric, Maria CS Inacio, Robert S Namba

**Affiliations:** ^1^Southern California Permanente Medical Group, Department of Orthopedic Surgery, Kaiser Permanente, Los Angeles, CA; ^2^Department of Surgical Outcomes and Analysis,Kaiser Permanente, San Diego, CA; ^3^Southern California Permanente Medical Group, Department of Orthopedic Surgery, Kaiser Permanente, Irvine, CA, USA.

## Abstract

**Background and purpose:**

The prevalence of chronic renal disease (CRD) is rising worldwide. Patients with CRD are more likely to have associated medical problems and are at greater risk of postoperative morbidity and mortality. We evaluated patient characteristics and risk of early revision, surgical site infection (SSI), thromboembolic events, mortality, and re-admission of patients with CRD undergoing total knee arthroplasty (TKA). We hypothesized that this patient population would have higher rates of complications.

**Patients and methods:**

We conducted a retrospective analysis of data that had been prospectively collected by a Total Joint Replacement Registry. All primary TKAs performed from 2005 through 2010 were included. 41,852 primary TKA cases were evaluated, of which 2,686 (6.4%) TKA procedures had been performed in CRD patients. Patient characteristics, comorbidities, and general health status were evaluated. Cox proportional hazard regressions and logistic regressions were used to evaluate the association of CRD with outcomes while adjusting for confounding variables.

**Results:**

The mean age of the CRD cohort was 67 years and approximately two-thirds of the patients were female. The median follow-up time was 2.1 years. Compared to TKA patients without CRD the CRD patients were older, had poorer general health, and had a higher prevalence of comorbidities. They had a higher incidence of deep SSI (0.9% vs. 0.7%), superficial SSI (0.5% vs. 0.3%), deep vein thrombosis (0.6% vs. 0.4%), any-time mortality (4.7% vs. 2.4%), 90-day mortality (0.4% vs. 0.2%), and 90-day re-admission (10% vs. 6.0%) than patients without CRD. However, after adjustment for confounding variables, CRD patients were at 1.9 times (95% CI: 1.1–3.5) increased risk of superficial SSI, 1.3 times (CI: 1.1–1.6) increased risk of re-admission within 90 days, and 1.5 times (CI: 1.2–1.8) increased risk of mortality at any point after the procedure. The risks of all other complications were not statistically significantly different in patients with CRD compared to patients without CRD.

**Conclusions:**

CRD patients undergoing TKA have more comorbidities and a higher risk for superficial SSI, 90-day re-admission, and any-time mortality.

The Centers for Disease Control and Prevention (CDC) has estimated that the prevalence of chronic renal disease (CRD) is 35% in adults with diabetes and greater than 40% in individuals over the age of 60 (Collins et al. 2011). The degree of renal dysfunction in CRD is graded in 5 stages. Stages 3–5 represent individuals who have lost at least half of normal adult renal function. Impaired renal function of this degree is related to the dysfunction of multiple organ systems, and its effect on the health of an individual is well documented (CDC 2010, Collins et al. 2011). This detrimental effect has been manifested in a growing percentage of the population, with the number of individuals suffering from end-stage renal disease (ESRD) rising by 600% over the last 30 years (CDC 2010).

In studies that have investigated outcomes of arthroplasty in individuals with ESRD (Lieberman et al. 1995, Sunday et al. 2002, Shrader et al. 2006, Debarge et al. 2007, Garcia-Ramiro et al. 2008), higher mortality rates, infection rates, and revision rates were reported. However, these studies focused on total hip arthroplasty (THA), and ESRD patients constitute only 3% of all patients with chronic renal insufficiency (Collins et al. 2011). Patients with ESRD, the most advanced form of CRD, are usually in need of kidney replacement therapy (dialysis or transplantation). To our knowledge, no published studies have examined the effects of CRD on total knee arthroplasty (TKA) when performed in a population of individuals suffering from a wide range of stages of renal dysfunction.

There is evidence to suggest that less advanced forms of the disease may have an appreciable effect on individuals undergoing TKA surgery. Patients with CRD are more likely to have associated medical problems (Go et al. 2004), the disease is a risk factor for postoperative complications (Shorr et al. 2012), and the impact of renal impairment on patients undergoing orthopedic procedures has been documented (Lejus et al. 2012, Pumberger et al. 2012). Furthermore, arthroplasty patients have previously been shown to have reduced renal function as they recover from this surgery (Nergelius et al. 1997).

Here we describe patient characteristics and surgical outcomes of patients with CRD who underwent TKA, and we evaluate association of CRD with the surgical outcomes of TKA procedures in a community-based sample of patients. We hypothesized that CRD patients undergoing TKA would have higher rates of perioperative complications, of re-admission, and of early implant revision.

## Patients and methods

### Study design and study sample

We performed a retrospective analysis of prospectively collected data. All primary elective TKAs performed for any diagnosis in 2 large geographical regions (Southern and Northern California) and entered into a Total Joint Replacement Registry (TJRR) between January 1, 2005 and December 31, 2010 were selected for the study. Revision, unicompartmental, and conversion knee arthroplasty procedures were excluded from the sample.

The TJRR covers a population of 9.2 million members of an integrated healthcare system (Kaiser Permanente) throughout 7 geographical regions in the USA; in 2010, it had a voluntary participation rate of 95% for primary and revision TKA procedures (Paxton et al. 2012). This integrated healthcare system is both the insurance provider and the healthcare delivery system for all patients. All inpatient, outpatient, laboratory, and pharmacy services are delivered by institutions within this healthcare system. The 2 largest TJRR-participating regions (involving 33 hospitals and 231 surgeons) were included in the study sample due to the availability of the data required for the study.

All the data used in this study were obtained from the TJRR. The data are collected, managed, and validated quarterly by the TJRR, and an analytical file is released on an annual basis. Data collection procedures, identification of complications and surgical outcomes, and validation procedures used by the TJRR have been published previously (Paxton et al. 2012). Briefly, all intraoperative information for the TJRR is collected by the surgeon at the time of the procedure and sent to the data repository, where it is entered manually. Patient and surgical information is enhanced using the electronic medical records (EMR) and other administrative (e.g. claims) and clinical databases (e.g. diabetes registries). The EMR is searched using diagnostic and procedural ICD 9 codes, specific parameters (using text searches), and certain condition indicators depending on the location of the information extracted. Information regarding revisions, SSI, and thromboembolic complications is collected using reporting from surgeons and through an active search for complications using the EMR available at the institution, and is adjudicated by trained clinical content experts. The algorithm and the screening process used to identify SSI have high sensitivity and specificity (Inacio et al. 2011). Mortality information is obtained from the EMR and the membership department within the institution, which is regularly updated by the Social Security Administration. Re-admission data are collected from the EMR using all inpatient admissions. All record linkages are done using unique patient identifiers employed by the institution.

### Exposure of interest

The main exposure of interest in this study was CRD. CRD in the sample identified was determined using the Elixhauser co-morbidity algorithm (HCUP 2012). Briefly, a combination of International Classification of Diseases (ICD) Ninth Revision codes (585.3, 585.4, 585.5, 585.6, 585.9, 586, V42.0, V45.1, V56.0–V56.32, V56.8, V45.11, V45.12, 403.01, 403.11, 403.91, 404.02, 404.12, 404.92, 404.03, 404.13, 404.93) and diagnosis-related groups (DRG) (652, 656-661, 673-675, 682-700, 652, 682-685) were used to identify patients with CRD at the time of surgery (and not complications due to kidney problems). The ICD 9 codes within the algorithm were also employed to identify and categorize all the patients who had been diagnosed with stage-3 (585.3), stage-4 (585.4), or stage-5 CRD (585.5, 585.6, 403.01, 403.11, 403.91, 404.02, 404.12, 404.92, 404.03, 404.13, 404.93). Patients had been previously diagnosed with CRD based on glomerular filtration rates estimated from serial serum creatinine values.

### Outcomes of interest

Implant survivorship (revision), postoperative complications (deep vein thrombosis (DVT), pulmonary embolism (PE), infection), length of stay, re-admission, and mortality were evaluated. Revision was defined as any reoperation in the same knee at any time after the index primary procedure during which a component was removed or replaced. SSIs were adjudicated using medical charts and categorized using a modified version of the CDC/National Healthcare Safety Network definitions, as organ/space SSIs are grouped together with deep incisional SSIs and termed deep SSI (Horan et al. 2008). A superficial SSI can occur within 30 days of the procedure, while a deep SSI can occur within 1 year of the procedure. DVT and PE (within 90 days of the index procedure) were identified using the Agency for Healthcare Research and Quality (AHRQ) Patient Safety Indicators and confirmed using EMR review. Re-admission was defined as any subsequent hospitalization (inpatient encounter only) within 90 days of the index procedure discharge date. Mortality was categorized into postoperative deaths occurring within 30 days, within 90 days, or at any time after surgery.

### Covariates

Patient characteristics (age, sex, BMI, race/ethnicity, and diagnosis for TKA), patient comorbidities (diabetes status, congestive heart failure, valvular disease, peripheral vascular disease, alcohol abuse, hypertension), and general health status (as measured by the American Society of Anesthesiologist (ASA) score) for the sample were evaluated and included in the analysis if they were considered confounders of the association between CRD and outcomes. Confounding variables were selected based on clinical relevance and availability through the registry. The operative time and length of hospital stay were also evaluated.

### Statistics

Frequencies, proportions, means, standard deviations (SDs), medians, and interquartile ranges (IQRs) were used to describe the study sample. When comparing patients with CRD to those without CRD, chi-square tests were used for categorical variables and Student t-tests were used for continuous variables. The incidence of crude revision, SSI, DVT, PE, mortality (30 day, 90 day, and overall), and 90-day re-admission were calculated for patients with CRD and for patients without CRD. Re-admission and length of stay data were available for a smaller patient sample within the study (January 1, 2009 to December 31, 2010); therefore, the denominator for these measures is different from the denominator used for the rest of the analysis. Binary logistic regression models were used to assess odds ratios (ORs) and 95% confidence intervals (CIs) of the likelihood of the non-time-dependent outcomes (septic revision, SSI, DVT, PE, 30- and 90-day mortality, and re-admission). Variable distributions were evaluated using histograms and residuals where appropriate.

Kaplan-Meier survival curves were used to describe the crude survival rates of the time-dependent outcomes (overall revision, aseptic revision, and mortality) and log-rank tests were used to compare survival distributions. Cox proportional hazard models were used to assess hazard ratios (HRs) and CIs for the risk of the time-dependent outcomes. Proportional hazard assumptions were evaluated using graphs of survival function against survival time. Cases were censored if they left the integrated healthcare system before the end of the study period, if they died, or if they had the event of interest. Covariates were explored as confounders of the association between CRD and likelihood of the non-time-dependent events and time-dependent events. Variables judged to be clinically important were included in the final models. Final models were adjusted for age, sex, race/ethnicity, ASA score, surgery indication (osteoarthritis vs. other), and the following comorbidities: diabetes, congestive heart failure, valvular disease, peripheral vascular disease, alcohol abuse, and hypertension. The study sample was missing CRD data on 4,970 (11.9%) cases. All cases with missing data were compared to cases with complete data to evaluate whether missing data biased our estimations. To handle these missing data and other missing data in our sample, we performed multiple imputations to create 10 versions of the analytical dataset and then used Rubin’s (1987) combining rules to calculate the final parameter estimates and confidence intervals from the 10 output sets. The imputation model used included all other covariates as well as the event indicator. Models using only completed cases were employed to examine consistency and robustness of the estimations of models with imputed data (Appendix A).

Data were analyzed using SAS version 9.2 and p < 0.05 was used as the threshold for statistical significance.

### Ethics

Internal Review Board (IRB # 5488) approval was obtained before commencement of the study.

## Results

The study sample consisted of 41,852 primary TKA cases. The median follow-up time for the cohort was 2.1 (0–5) years. 6.4% (n = 2,686) of the TKAs were performed in patients with CRD. Of these 2,686 cases, 69% (n = 1,843) had been diagnosed with CRD stage 3, 3.0% (n = 80) with stage 4, and 5.5% (n = 148) with stage 5. In addition, 0.1% (n = 3) had been diagnosed as CRD “other,” 0.9% (n = 25) had had a previous kidney transplant, and 22% (n = 587) had been diagnosed as CRD “unspecified.”

Compared to patients without CRD, patients with CRD had a higher proportion of males (40% vs. 37%), of blacks (13% vs. 9.1%), of patients with ASA scores ≥ 3 (66% vs. 39%), and they had a higher prevalence of diabetes (46% vs. 25%), congestive heart failure (12% vs. 2.6%), valvular disease (6.5% vs. 2.8%), peripheral vascular disease (10% vs. 3.1%), alcohol abuse (1.8% vs. 1.3%), and hypertension (95% vs. 63%). The median age of the TKA patients with CRD was also higher (73, range 39–97) than that of the TKA patients without CRD (67, range 19–101). The median operative time was 90 min for both groups; the ranges for both groups differed slightly. The median length of stay for the TKA procedure was 3 days in both groups (Table 1).

**Table 1.** Primary total knee arthroplasty samples included in the study, overall and according to chronic renal disease status, 2005–2010

	Chronic renal disease status								
	Total		No		Yes		p-value	Unknown	
	n = 41,852		n = 34,196 (81.7%)		n = 2,686 (6.4%)			n = 4,970 (11.9%)	
	n	%	n	%	n	%		n	%
Sex									
Female	26,058	62.3	21,420	62.6	1,607	59.8	0.004	3,031	61.0
Male	15,785	37.7	12,769	37.3	1,079	40.2		1,937	39.0
Unknown	9	0.0	7	0.0	0	0.0		2	0.0
Race/ethnicity									
White	28,307	67.6	23,192	67.8	1,796	66.9	< 0.001	3,319	66.8
Hispanic	6,080	14.5	5,190	15.2	296	11.0		594	12.0
Black	3,786	9.1	3,098	9.1	347	12.9		341	6.9
Asian	2,241	5.4	1,918	5.6	186	6.9		137	2.8
Other/Multi	740	1.8	578	1.7	60	2.2		102	2.1
Unknown	698	1.7	220	0.6	1	0.0		477	9.6
ASA category									
1 and 2	24,401	58.3	20,749	60.7	909	33.8	< 0.001	2,743	55.2
≥ 3	16,855	40.3	13,378	39.1	1,764	65.7		1,713	34.5
Unknown	596	1.4	69	0.2	13	0.5		514	10.3
Comorbidities									
Diabetes	11,007	26.3	8,567	25.1	1,231	45.8	< 0.001	1,209	24.3
Congestive heart failure	1,203	2.9	894	2.6	309	11.5	< 0.001	–	–
Valvular disease	1,127	2.7	952	2.8	175	6.5	< 0.001	–	–
Peripheral vascular disease	1,326	3.2	1,051	3.1	275	10.2	< 0.001	–	–
Alcohol abuse	476	1.1	429	1.3	47	1.8	0.03	–	–
Hypertension	23,991	57.3	21,450	62.7	2,541	94.6	< 0.001	–	–
Diagnosis									
Osteoarthritis	40,773	97.4	33,271	97.3	2,645	98.5	0.002	4,857	97.7
Osteonecrosis	162	0.4	136	0.4	7	0.3	0.3	19	0.4
Posttraumatic arthritis	430	1.0	362	1.1	13	0.5	0.004	55	1.1
Rheumatoid arthritis	814	1.9	685	2.0	39	1.5	0.05	90	1.8
	Median	Range	Median	Range	Median	Range		Median	Range
Age **^a^**	67	11–101	67	19–101	73	39–97	< 0.001	66	11–98
BMI, kg/m**^2^** **^a^**	30.8	15.4–68.5	30.7	15.4–68.5	30.7	15.7–56.7	0.8	31.4	15.4–65.9
Operative time, min **^a^**	90	30–480	90	30–480	90	30–369	0.02	80	30–300
Length of stay **^b^**	3	0–33	3	0–33	3	1–24	< 0.001	3	2–8

BMI: body mass index; ASA: American Society of Anaesthesiologists score.

**^a^** Unknown: age < 0.1%, BMI 0.4%, operative time 18%.

**^b^** Length of stay available for limited sample, 2009 onwards (n = 18,821). Unknown: 13%.

**Figure 1.**
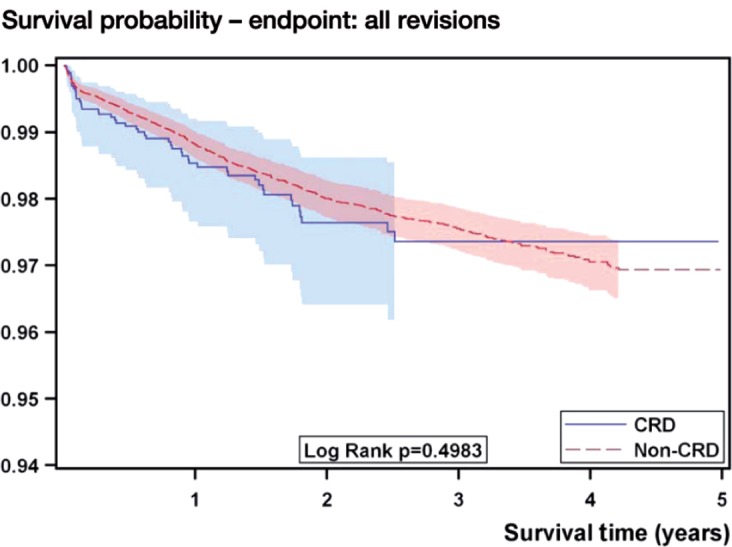
Kaplan-Meier survival estimates for TKA, with 95% confidence limits, according to whether or not the patients had chronic renal disease (CRD). Overall revisions. (N = 36,882, CRD = 2,682 and non-CRD = 34,196).

**Figure 2.**
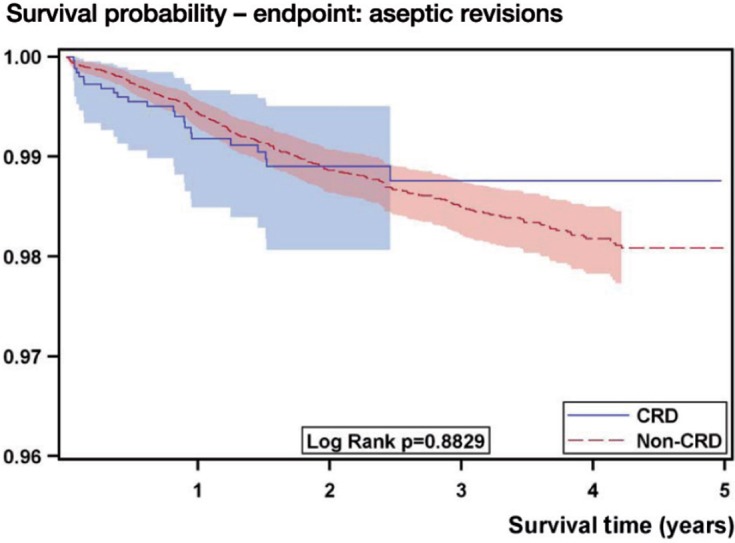
Kaplan-Meier survival estimates for TKA, with 95% confidence limits, according to whether or not the patients had chronic renal disease (CRD). Aseptic revisions only. (N = 36,574, CRD = 2,661 and non-CRD = 33,913).

**Figure 3.**
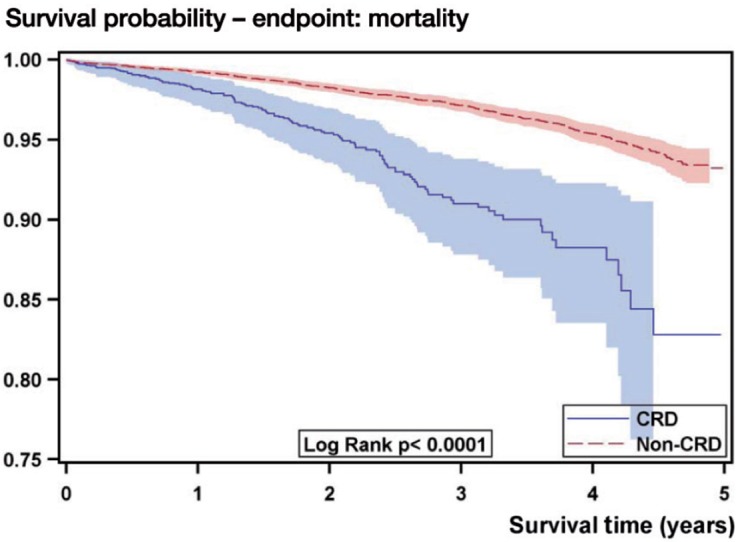
Kaplan-Meier survival estimates for TKA, with 95% confidence limits, according to whether or not the patients had chronic renal disease (CRD). Mortality. (N = 36,882, CRD = 2,682 and non-CRD = 34,196).

Patients with CRD had a higher crude incidence for most of the outcomes evaluated (Table 2). TKAs in CRD patients had a higher crude incidence of deep SSI (0.89% vs. 0.70%), superficial SSI (0.52% vs. 0.31%), DVT (0.56% vs. 0.42%), any-time mortality (4.7% vs. 2.4%), 90-day mortality (0.41% vs. 0.24%), and 90-day re-admission (10% vs. 6.0%). Of the patients who did not undergo a revision, 3.1% were lost to follow-up during the study period (Figures 1–3).

**Table 2.** Frequencies and proportions of surgical outcomes for the total cohort and according to chronic renal disease status, 2005–2010

	Chronic renal disease status							
	Total		No		Yes		Unknown	
	n = 41,852		n = 34,196		n = 2,686		n = 4,970	
	n	%	n	%	n	%	n	%
Revision (any)	792	1.89	660	1.93	48	1.79	84	1.69
Septic revision	340	0.81	283	0.83	25	0.93	32	0.64
Aseptic revision	452	1.08	377	1.10	23	0.86	52	1.05
Surgical site infection (deep)	297	0.71	241	0.70	24	0.89	32	0.64
Surgical site infection (superficial)	130	0.31	106	0.31	14	0.52	10	0.20
Pulmonary embolism	211	0.50	175	0.51	15	0.56	21	0.42
Deep vein thrombosis	184	0.44	142	0.42	15	0.56	27	0.54
Mortality (any time)	1,043	2.49	827	2.42	125	4.65	91	1.83
Mortality within 30 days	52	0.12	46	0.13	4	0.15	2	0.04
Mortality within 90 days	101	0.24	81	0.24	11	0.41	9	0.18
Re-admission within 90 days **^a^**	1,053	5.59	889	5.98	164	10.35	–	–

**^a^** Limited sample for re-admission data, 2009 onwards (n = 18,821). Unknown: 13%.

Controlling for confounding variables reduced the number of statistically significant differences in outcome between TKA procedures performed in patients with CRD and TKA procedures in patients without CRD (Table 3). After adjustment for age, sex, ASA score, indication for surgery, and comorbidities, the risks of early revision overall, early aseptic revision, septic revision, deep SSI, DVT, PE, 30-day mortality, and 90-day mortality were not statistically significant. The risks of any-time mortality (HR = 1.5, 95% CI: 1.2–1.8), 90-day re-admission (OR = 1.3, 95% CI: 1.1–1.9), and superficial SSI (OR = 1.9, 95% CI: 1.1–3.5) were higher for patients with CRD than for those without CRD. All risk estimations from models employing only complete data were consistent with multiple imputation models (see Appendix, Supplementary data).

**Table 3.** Crude and adjusted associations of outcomes in TKA cases with chronic kidney disease compared to TKA cases without chronic renal disease, 2005–2010 (n = 41,852)

	Crude HR/OR	95% CI	p-value	Adjusted **^a^** HR/OR	95% CI	p-value
Hazard ratio – Time-dependent						
Revision (any)	1.07	0.80–1.44	0.6	1.04	0.77–1.41	0.8
Aseptic revision	0.90	0.60–1.39	0.7	1.05	0.69–1.61	0.8
Mortality (any time)	2.76	2.28–3.35	< 0.001	1.49	1.21–1.83	< 0.001
Odds ratio – Not time-dependent						
Septic revision	1.13	0.75–1.72	0.6	0.90	0.59–1.38	0.6
Surgical site infection (deep)	1.26	0.82–1.93	0.3	1.01	0.65–1.58	1.0
Surgical site infection (superficial)	1.73	0.98–3.06	0.06	1.92	1.05–3.50	0.04
Pulmonary embolism	1.10	0.64–1.88	0.7	0.91	0.52–1.60	0.7
Deep vein thrombosis	1.38	0.80–2.37	0.2	0.95	0.54–1.68	0.9
Mortality within 30 days	1.17	0.43–3.18	0.8	0.59	0.21–1.67	0.3
Mortality within 90 days	1.81	0.97–3.37	0.06	0.88	0.45–1.70	0.7
Re-admission within 90 days **^b^**	1.81	1.52–2.16	< 0.001	1.33	1.11–1.61	0.003

HR: hazard ratio; OR: odds ratio.

**^a^** Models adjusted for age, sex, race/ethnicity, American Society of Anaesthesiologists scores, surgery indication (osteoarthritis vs. other diagnosis), and comorbidities (diabetes, heart failure, valvular disease, peripheral vascu- lar disease, alcohol abuse, and hypertension).

**^b^** Limited sample for re-admission data, 2009 onwards (n = 18,821).

Of the 1,568 CRD TKA patients for whom re-admission data were available, there were 164 re-admissions within 90 days of the procedure (11%). Because some patients had more than 1 admission diagnosis, 237 admission diagnoses were listed for these patients. Of these 237 admission diagnoses, the 3 most common were acute renal failure (4.6%), gastrointestinal Clostridium difficile infection (3.8%), and deep infection of the total knee prosthesis (3.8%). Grouping of admission diagnoses according to organ system revealed that the most commonly affected systems were gastrointestinal (15%), cardiac (8.9%), and surgical site/prosthesis (8.4%).

In the 14,860 non-CRD TKA patients for whom re-admission data were available, there were 889 (6.0%) re-admissions within 90 days of the procedure. 1,184 admission diagnoses were listed for these patients. Among these 1,184 diagnoses, the 3 most common were postoperative wound infection (6.2%), deep infection of the prosthesis (4.3%), and non-infection-related complication of prosthesis (2.9%). Grouping of admission diagnoses revealed that complications involving the surgical site and prosthesis were the most common (24%).

## Discussion

Unlike the previous literature, when comparing patients with CRD to those without the disease we found few differences in TKA-related perioperative morbidity and no differences in early implant revision rates. There were no significant differences in rates of deep SSI, DVT, PE, 30-day mortality, or 90-day mortality. Significant differences between the 2 groups were limited to superficial SSI, 90-day re-admission, and mortality at any point after TKA in patients with CRD.

Most published studies have reported greater rates of mortality, revision, infection, and other morbidity when total joint arthroplasty is performed in patients with renal impairment (Lieberman et al. 1995, Sunday et al. 2002, Shrader et al. 2006, Debarge et al. 2007, Garcia-Ramiro et al. 2008). However, these studies focused on THA performed in patients with ESRD. McCleery et al. (2012) reported an increase in rates of TKA-related infection and revision in patients with renal failure, patients on hemodialysis, and patients after renal transplant. Patients with renal failure had an increased risk of early and late infection. Those on renal dialysis had higher risks of late infection and early revision. Also, renal transplant patients had a higher risk of late infection. The authors postulated that there was a dose-dependent relationship between disease severity and outcome of TKA. Furthermore, they recommended that a future study should assess TKA outcomes in patients with a broader range of disease severity. The nature of our data source (a registry) allowed an evaluation of patients with just such a broader range of disease severity.

Since individuals with CRD have usually been diagnosed with other diseases that can affect surgical outcome (Go et al. 2004, Chauhan and Vaid 2009), it is possible that the results of TKA would be adversely affected by the presence of CRD in patients. Indeed, our CRD sample had a higher prevalence of multiple comorbidities—including diabetes, hypertension, peripheral vascular disease, cardiac disease, and alcohol abuse. CRD has been cited as a risk factor for increased mortality, infection, and other morbidity (Bozic et al. 2012a, b, Shorr et al. 2012, Rodriguez-Merchen 2012) and individuals with CRD reportedly have a higher complication rate when undergoing a variety of orthopedic procedures (Lejus et al. 2012, Pumberger et al. 2012). Patients with TKA may be further affected, as CRD has been noted to have a deleterious effect on the anatomic structures in and around the knee (Muratli et al. 2005).

There are a variety of reasons why our results differed from those of previous studies. First, we expanded the definition of CRD to include a larger sample of patients. The previous focus on ESRD (stage 5) excluded the vast majority of individuals with CRD who had milder forms of the disease. We elected to include patients at stages 3 and 4 in an effort to describe surgical outcome in a broader patient sample. Although CRD at stages 3 and 4 remains clinically significant, it has less of an effect on patient health (Go et al. 2004, Rodriguez-Merchen 2012). Inclusion of TKA patients with stage-3 and stage-4 CRD produced a study sample for which most complication rates were similar to those of the general population.

Secondly, we consider that the improved TKA outcomes in our patient sample may also have been due to advances in patient care since the earlier studies. Our patient sample underwent primary TKA from 2005 to 2010. Many of the earlier studies followed patients who had undergone surgery many years, even decades, earlier. Advances in testing, treatment, and surgical technique may have contributed to reduced perioperative morbidity. We base this on the fact that the stage-5 CRD patients in this study experienced relatively low complication rates in comparison to ESRD patients in previous studies. The 148 individuals with stage-5 CRD experienced relatively low rates of infection (2.0%), revision (1.4%), any-time mortality (19%), and all other morbidity (0.7%). The small number of patients in this group prevented evaluation of the effects of CRD stage and the effects of dialysis. However, it may be reasonable to compare the stage-5 results in this study to previously published results from studies with similar numbers of study subjects. Almost all previous studies of TJAs performed in ESRD patients have reported greatly elevated rates of infection (13–33%), revision (22–36%), mortality (17–58%), and other morbidity (29–74%) (Lieberman et al. 1995, Sunday et al. 2002, Shrader et al. 2006, Debarge et al. 2007, Garcia-Ramiro et al. 2008). The more recent study by McCleery et al. (2012) stands as an exception, and with lower complication rates in patients on dialysis. While these complication rates remain greater than those in our study, the trend of decreasing complication rates might suggest that improvements in patient care have a bearing on patient outcome.

Despite the similarity in patient outcome found in our study between patients with and without CRD, we did find 3 statistically significant differences. Patients with CRD were more likely to have a superficial SSI, re-admission within 90 days, and mortality during the entire follow-up period. Some confounding variables contribute to these postoperative results, however, the presence of CRD is itself a significant risk factor.

With the exception of superficial SSIs, patients with and without CRD may experience a similar outcome immediately after TKA. Yet those with CRD remain a fundamentally different patient population. They are more likely to have serious associated medical conditions, which may be a factor in the subsequent re-admission rate. In patients without CRD, the 3 most common re-admission diagnoses were all related to the surgical site or prosthesis: postoperative wound infection, infection of the prosthesis, and non-infectious complication of the prosthesis. In contrast, in patients with CRD, 2 of the top 3 re-admission diagnoses were medical and unrelated to the surgical site or prothesis: acute renal failure, gastrointestinal infection, and infection of the prosthesis.

Analysis of the re-admission diagnoses by system type further illustrates the difference in distribution between the 2 study groups. In patients without CRD, a complication involving the prosthesis or surgical site was the most common reason for re-admission, and accounted for 24% of all re-admission diagnoses. In contrast, in patients with CRD, a complication involving the prosthesis or surgical site was the third most common reason for re-admission. Gastrointestinal and cardiac complications were the 2 most common. Anticipation of potential re-admissions and focusing on these top re-admission diagnoses may help to reduce 90-day re-admission and mortality rates.

Due to the small sample sizes of patients with ESRD and patients undergoing dialysis, we are limited in our ability to draw conclusions regarding the outcomes of TKA among patients in these two groups. Furthermore, the sample size of the CRD cohort may still not have been enough for us to be able to detect small effect outcomes such as 30-day and 90-day mortality. We were also limited by the short-term follow-up of our cohort and cannot therefore only evaluate the risk of the time-dependent events for the period described. In addition, the lack of clinical data limits our ability to comment on the effect of CRD on the clinical outcome of TKA.

Our study was further limited by the use of the Elixhauser co-morbidity algorithm to identify and categorize patients with CRD, which may have led to misclassification in a small proportion of these cases. However, this algorithm has been validated and has been already employed in many epidemiological studies. Also, information regarding CRD status was incomplete for a proportion of the study sample. This could lead to informational bias. However, we addressed this by using multiple imputation techniques and by creating complete data analysis models to verify the robustness and consistency of our findings. Despite these limitations, 2 recent studies have suggested that the estimated CRD prevalence of 6.4% in our sample is reasonable. Coresh et al. (2007) reported a prevalence of CRD of 8.1% in the United States for 1999–2004, and Cram et al. (2012) reported a CRD prevalence of 4.1% in Medicare patients who underwent primary TKA between 2007 and 2010.

The strengths of the present study include the large and representative sample of TKA cases (Karter et al. 2002, Koebnick et al. 2012) and the study setting (community-based practices). This patient sample and the large number of contributing surgeons, with different surgical volumes, different hospital settings, and varying degrees of experience, increase the generalizibility of our results. The use of prospectively collected data and validated algorithms is another strength of our study. Furthermore, the large sample size enabled us to establish the effect of confounding variables in the relationship between CRD and TKA outcomes. With the inclusion of patients with a broader array of severity of renal disease, we feel that our study presents a more accurate depiction of TKA outcome in all patients with CRD. This procedure can be performed relatively safely in this patient population, with early revision rates, morbidity rates, and mortality rates far below those previously reported. However, one cannot ignore that these patients do present with a wide variety of medical problems and require close attention both during and well beyond the perioperative period.

### Supplementary data

Appendix is available at Acta’s website (www.actaorthop.org), identification number 6425.
