# Supplementary material for: Can total knee arthroplasty be safely performed in patients with chronic renal disease? An evaluation of perioperative morbidity in 2,686 procedures from a Total Joint Replacement Registry
Source: Acta Orthop. 2014 Feb 25;85(1):71–8. doi: 10.3109/17453674.2013.878829 (PMC3940995; doi:10.3109/17453674.2013.878829)
Supplement: Supplementary file 1 [file ORT-85-71-s6425.pdf]

Supplementary article data

Can total knee arthroplasty be safely performed in patients with chronic renal disease?

An evaluation of perioperative morbidity in 2,686 procedures from a Total Joint Replacement Registry

Alexander Miric[1], Maria CS Inacio[2], and Robert S Namba[3]

[1]Southern California Permanente Medical Group, Department of Orthopedic Surgery, Kaiser Permanente, Los Angeles, CA; [2]Department of Surgical Outcomes and Analysis, Kaiser Permanente, San Diego, CA; [3]Southern California Permanente Medical Group, Department of Orthopedic Surgery, Kaiser Permanente, Irvine, CA, USA.
Correspondence: alex.x.miric@kp.org
Submitted 13-04-30. Accepted 13-11-08

Appendix. Crude and adjusted associations of outcomes in TKA cases with chronic kidney disease compared to cases without chronic renal disease, 2005–2010 (n = 36,872; complete data only)

	Crude HR/OR	95% CI	p-value	Adjusted [a] HR/OR	95% CI	p-value
Hazard ratio – Time-dependent						
Revision (any)	1.11	0.83–1.48	0.5	1.07	0.79–1.46	0.7
Aseptic revision	0.97	0.64–1.47	0.9	1.14	0.74–1.75	0.6
Mortality (any time)	2.81	2.33–3.40	< 0.001	1.50	1.23–1.85	< 0.001
Odds ratio – Not time-dependent						
Septic revision	1.13	0.75–1.70	0.6	0.89	0.58–1.37	0.6
Surgical site infection (deep)	1.40	1.00–1.96	0.05	1.23	0.86–1.75	0.3
Surgical site infection (superficial)	1.69	0.96–2.95	0.07	1.85	1.03–3.35	0.04
Pulmonary embolism	1.09	0.64–1.85	0.7	0.91	0.52–1.57	0.7
Deep vein thrombosis	1.35	0.79–2.30	0.3	0.94	0.54–1.65	0.9
Mortality within 30 days	1.11	0.40–3.08	0.8	0.56	0.19–1.63	0.3
Mortality within 90 days	1.73	0.92–3.26	0.09	0.85	0.43–1.65	0.6
Re-admission within 90 days [b]	1.81	1.52–2.16	< 0.001	1.34	1.11–1.61	0.003

HR: hazard ratio; OR: odds ratio.
[a] Models adjusted for age, sex, ethnicity, American Society of Anaesthesiologists scores, surgery indication (osteoarthritis vs. other diagnosis), and comorbidities (diabetes, heart failure, valvular disease, peripheral vascular disease, alcohol abuse, and hypertension).
[b] Limited sample for re-admission data, 2009 onwards (n = 16,419; complete data only).

Open Access - This article is distributed under the terms of the Creative Commons Attribution Noncommercial License which permits any noncommercial use, distribution, and reproduction in any medium, provided the source is credited.
DOI 10.3109/17453674.2013.878829
